# Satisfaction of psychiatric inpatients in China: clinical and institutional correlates in a national sample

**DOI:** 10.1186/s12888-019-2011-0

**Published:** 2019-01-11

**Authors:** Feng Jiang, Jeffrey Rakofsky, Huixuan Zhou, Linlin Hu, Tingfang Liu, Shichao Wu, Pengyu Zhao, Huanzhong Liu, Yuanli Liu, Yi-lang Tang

**Affiliations:** 1 School of public health, Chinese Academy of Medical Sciences and Peking Union Medical College, No.3 Dong Dan San Tiao, Dongcheng District, Beijing, China; 2 Department of Psychiatry and Behavioral Sciences, Emory University, 12 Executive Park Drive NE, Suite 300, Atlanta, GA USA; 3 Institute for Hospital Management of Tsinghua University, No.30 Shuang Qing Road, Haidian District, Beijing, China; 4 Department of Psychiatry, Chaohu Hospital of Anhui Medical University, No. 64 Chaohu Road, Hefei, China; 5 Atlanta VA Medical Center, 1670 Clairmont Road, Decatur, GA USA

## Abstract

**Background:**

Surveying patients’ satisfaction is essential to improve patient-centered care, however, studies on satisfaction and their correlates among psychiatric inpatients are rare in China. This study aimed to measure satisfaction levels of psychiatric inpatients in a national sample and to examine individual and institutional correlates.

**Methods:**

As part of the National Survey for the Evaluation of Psychiatric Hospital Performance, psychiatric inpatients from 32 tertiary psychiatric hospitals in 29 Chinese provinces were interviewed on the day of discharge by trained research staff. Satisfaction was assessed using a five-item questionnaire. Patients’ sociodemographic and clinical information were manually retrieved from medical records and institutional data were provided by participating hospitals. Multilevel linear regression was used to assess factors associated with level of satisfaction.

**Results:**

Among 1663 inpatients, the reported satisfaction levels were high, with a mean score of 23.3 ± 2.4 out of 25. Education level was positively associated with global satisfaction, satisfaction with costs, and satisfaction with privacy protection. Treatment response was associated with global satisfaction and with the doctor-patient communication subscore. The number of psychotherapy sessions was positively associated with the privacy protection subscore (coefficient = 0.0, *P* = 0.046). The Global Assessment of Function score was positively associated with the doctor-patient communication subscore (coefficient = 0.0, *P* = 0.003). Total satisfaction scores and all five subscores were positively associated with hospital-level factors, and patients discharged from hospitals with better staffing and resources reported significantly higher levels of satisfaction.

**Conclusion:**

Overall, psychiatric inpatients in China were satisfied with the services they received. To further improve patient satisfaction, mental health professionals should optimize their patients’ treatment response as much as possible before discharge and provide more psychological treatment during the hospitalization. The government should also provide more resources to increase the number of mental health professionals (nurses, psychologists, and psychiatrists) working in psychiatric hospitals.

## Background

Patient satisfaction is an important and commonly utilized measure for healthcare quality and research [1, 2]. Most theories and formulations about patient satisfaction are based on marketing theories. Generally, patient satisfaction is defined as the extent to which health services meet patients’ expectations [3]. Patient satisfaction is relevant to clinical medicine as higher levels of it can lead to better adherence with treatment recommendations [4]. Understanding the determinants of patient satisfaction can help improve patient satisfaction, which can in turn improve clinical outcomes, patient retention, rehospitalization [5–7], patient complaints, and lawsuits [8].

Many factors have been associated with patient satisfaction in psychiatry. A study conducted in the U.S. in the early 1980’s found that high quality medical staff was the greatest source of satisfaction, while the cost of care was associated with the most dissatisfaction in a private hospital [9]. Another study in the 1990’s found nurses were seen as the most helpful healthcare professionals patients encountered [10]. While most surveys found psychiatric patient satisfaction was good or high [11, 12], and better than patients with non-psychiatric disorders, some found the contrary. In one study, the authors used survey data from the 1991 Medicare Current Beneficiary Survey. They found aged and disabled beneficiaries with psychiatric disorders were significantly less likely than those without disorders to be satisfied with the overall quality of health care, follow-up care, and the physician’s concern for their overall health [13]. With respect to psychiatric diagnosis, some studies found that patients with schizophrenia were less satisfied than patients with other psychiatric disorders [14–17], while other studies showed the opposite trend [11, 18]: patients with schizophrenia were more satisfied compared to those without schizophrenia.

Other factors associated with patient satisfaction have also been studied, including the provision of treatment information [12], psychiatrist-patient ratio [17], severity of disease and the number of prescribed drugs [16, 18], clinical improvement and seclusion [19], involuntary admission and functioning [20], coercive treatment, ward environment, and staff relationships [21, 22].

There have only been a few studies examining psychiatric patient satisfaction in China. Overall, the satisfaction levels of psychiatric patients in those studies were rather high. For example, a survey of 153 patients in a psychiatric hospital in Hebei province, found that the overall satisfaction score for nursing services was 89.5/100 [23]. Another survey, included 9718 discharged psychiatric patients in Liaoning province and found that the average satisfaction score was 99.2/100 [24]. Another similar survey included 4063 discharged patients from a tertiary psychiatric hospital in Hubei province and found that the satisfaction score was 95.7% [25]. Of note, all the above three studies were based in one local hospital and they all used a locally developed satisfaction scale. None of them reported correlates associated with patient satisfaction.

To address the gap in research, we conducted a nation-wide survey with the goal of determining the amount of satisfaction overall and for different aspects of the patients’ hospitalization. We also aimed to identify the clinical and institutional correlates with patient satisfaction levels.

## Methods

### Study design, setting and study sample

This study was a part of a larger research project, the National Survey for the Evaluation of Psychiatric Hospital Performance [26]. We selected one provincial psychiatric hospital under the jurisdiction of the Ministry of Health in the capital city of each province, except Beijing (where 3 were selected) and Anhui Province (2 were selected). Gansu and Tibet were not included because there were no psychiatric hospitals in their capital cities at the time of survey. Hospitals within the jurisdiction of the Ministry of Public Security (Forensic psychiatric hospitals) and the Ministry of Social Welfare (Safety net hospitals) were not selected. In total, 32 psychiatric hospitals from 29 provinces and autonomous regions in mainland China were selected. We included all psychiatric inpatients who were discharged from December 25 to 27, 2017.

The patients were interviewed by clinicians who were not directly involved in the patients’ care. Interviewers were trained on all of the aspects of the study protocol, including the research aims and how to use the study questionnaires. In this study, we included all adult patients who were older than 18 years old, and had stayed longer than 24 h and were discharged from hospitals from 25 to 27 December 2017. We excluded inpatients who were younger than 18 years old or those who stayed in hospitals less than one day. We also excluded patients who were missing one or more items on the satisfaction questionnaire.

### Patient satisfaction questionnaire

Currently, there were no international patient satisfaction scale available for psychiatric patients in Chinese language, so we developed a psychiatric inpatient satisfaction questionnaire for this study. Based on a literature review and expert opinions, three authors, FJ, YLT and HZL, developed the questionnaire. Then, a pilot study, which included 127 inpatients, was conducted to gather users’ feedback to improve the readability and reliability. Based on existing literature, the psychiatric inpatient satisfaction questionnaires have generally involved five domains: quality of care, interpersonal relations, costs of care, non-medical services, and global satisfaction [27]. The final version of our questionnaire was composed of the following questions: (1) How satisfied were you with the doctor-patient communication during your hospitalization? (communication subscore). (2) How satisfied were you with privacy-protection during your hospitalization? (privacy protection subscore). (3) How satisfied were you with medical services during your hospitalization? (medical services subscore). (4) How satisfied were you with hospitalization costs? (cost subscore). (5) How satisfied were you with your hospitalization overall? (global score).

All items were measured using the balanced 5-point Likert scale: very dissatisfied = 1, dissatisfied = 2, neutral = 3, satisfied = 4 and very satisfied = 5. The total score ranged from 5 (very dissatisfied for all items) to 25 (very satisfied for all items). The Cronbach’s α coefficient of the questionnaire was 0.90 and the test-retest reliability in the pilot study was 0.76.

To ensure confidentiality, psychiatric inpatients responded anonymously and items on personal identifiable information were kept to a minimum. A few open-ended questions were included, such as: what other comments/suggestions would you like to provide regarding this topic?

### Other study measures

Patients’ demographic information and clinical features were collected by site-based research staff using semi-structured interviews and discharge medical records. The retrieved data included age, sex, marital status, education level, primary clinical diagnosis according to *International Classification of Diseases and Related Health Problems 10th revision* (ICD-10) [28], the Global Assessment of Functioning (GAF) scale score at admission [29], and the treatment response measured by the Clinical Global Impression(CGI) scale [30]. Other data included days of hospitalization, number of psychological treatment sessions, physical restraint times, seclusion times, and number of electroconvulsive therapy (ECT) sessions. In China, the concept of psychological treatment is broad and inclusive. They often include individual therapy with either a psychiatrist or a psychologist (the number of social workers is very limited in psychiatric hospitals in China). They can also include group therapy led by a psychologist or sometimes a registered nurse. The commonly used approaches include psychoeducation, supportive, cognitive behavioral therapy, or dynamic approach. Number of psychological treatment sessions means the total number of psychological treatments the patients received. Hospital level data were retrieved from the Hospital Information System (HIS) and included the number of beds, physicians (including psychiatrists), nurses, and psychologists.

### Statistical analysis

Summary statistics were used to describe the data. Comparisons of total satisfaction score and dimension score in various subgroups were calculated using Mann-Whitney U test or Kruskal-Wallis test, as appropriate. Associations between patient satisfaction scores and the continuous variables were analyzed using Spearman’s correlation tests. The SPSS version 22.0 software package (SPSS Inc., Chicago, IL, USA) was used to perform the basic statistical analyses.

Because patients were nested in 32 hospitals, and because of the advantages of the multilevel model [31], this study applied multilevel linear regression with MLwiN 2.30 (Centre for Multilevel Modelling, University of Bristol, UK).

All of the tests were two-sided and statistical significance was defined as *P* < 0.05.

## Results

### Sample characteristics

One thousand seven hundred eighty psychiatric patients participated in the survey across 32 hospitals, 117 were excluded due to missing data, and 1663 were included in the final analysis. Patients characteristics are shown in Table 1. The mean age of participants was 41.9 years old, 51.7% were female, 52.6% were married, 26.1% had college or above education. 48.8% had a diagnosis of schizophrenia or related disorders, 43.1% were hospitalized psychiatrically for the first time, 48.9% were involuntarily admitted, 27.4% had received physical restraints during this hospital stay, 8.2% had received seclusion, and the median length of hospitalization stay was 32 days (interquartile range: 20–54 days). 57.5% were rated as having marked improvement on the day of discharge by their treating team.

**Table 1:** Sociodemographic and clinical characteristics of 1663 psychiatric inpatients

Characteristics	N (%)
Sociodemographic characteristics	
Sex	
Male	803 (48.3)
Female	860 (51.7)
Age (years, mean ± standard deviation)	41.9 ± 15.6
Marital status	
Married	874 (52.6)
Single or others	789 (47.4)
Education level	
Elementary school	267 (16.1)
Middle school	501 (30.1)
High school	461 (27.7)
College or above	434 (26.1)
Insurance coverage	
Self-pay	398 (23.9)
Others	1265 (76.1)
Hospitalization times	
First psychiatric hospitalization	717 (43.1)
Recurrent psychiatric admission	946 (56.9)
Involuntary hospitalization	
Yes	814 (48.9)
No	849 (51.1)
Physical restraints during hospital stay	
Yes	456 (27.4)
No	1207 (72.6)
Seclusion	
Yes	137 (8.2)
No	1526 (91.8)
Psychological treatment	
Yes	1539 (81.8)
No	303 (18.2)
ECT treatment^a^	
Yes	1422 (85.5)
No	241 (14.5)
Days of hospital stay, median (interquartile range)	32 (20–54)
Clinical characteristics	
Psychiatric diagnosis^c^	
Schizophrenia or related disorders	811 (48.8)
Mood disorders	527 (31.7)
Others	325 (19.5)
GAF (mean ± standard deviation) ^b^	46.2 ± 18.1
Treatment response	
Marked improvement	956 (57.5)
Improvement	623 (37.5)
Somewhat improvement	76 (4.6)
No change or worse	8 (0.5)

^a^ Electric convulsive treatment

^b^Global Assessment Functioning. The GAF assigns a clinical judgment to the individual’s overall functioning level and ranges from 0 (inadequate information) to 100 (superior functioning)

^c^ Psychiatric diagnosis defined by the International Classification of Diseases and Related Health Problems, Tenth Revision

Twelve hospitals were selected from East China, which is more population-dense and socio-economically developed, while 10 hospitals were from the less developed West China. Three hospitals were from Northeast China, and 7 hospitals were from Middle China. The hospital sizes varied, with bed number ranging from 169 to 2134 (median = 810). The doctor/bed ratios also varied, ranging from 0.1 to 0.3 (median = 0.2). The nurse/bed ratios ranged from 0.1 to 0.6 (median = 0.4), and psychologist/bed ratios ranged from 0 to 7.7% (median = 1.2%).

### Patients satisfaction

Total satisfaction scores were generally high across 32 participating hospitals, with a mean score of 23.3 ± 2.4 out of 25. Table 2 summarizes the total satisfaction scores by each hospital.

**Table 2:** Total satisfaction score by site (*N* = 32)

Hospital NO.	Participation	Mean	Standard Deviation
1	37	23.3	2.4
2	90	24.4	1.4
3	49	23.9	2.0
4	95	24.3	1.8
5	51	21.8	1.9
6	47	20.1	2.1
7	50	21.2	2.1
8	48	24.1	1.7
9	48	24.1	1.7
10	48	24.5	1.1
11	88	24.9	0.3
12	87	22.7	2.6
13	47	22.7	3.0
14	49	22.9	2.1
15	48	23.7	1.9
16	48	24.3	2.9
17	47	22.2	2.4
18	51	24.1	1.7
19	47	24.9	0.3
20	46	24.6	2.9
21	49	23.5	2.3
22	98	22.4	2.6
23	49	23.7	1.9
24	48	24.1	1.8
25	15	22.3	2.8
26	56	22.8	2.5
27	46	23.5	2.1
28	50	22.1	2.3
29	42	22.7	2.7
30	20	20.8	1.8
31	20	23.8	1.8
32	49	22.3	3.6
Total	1663	23.3	2.4

Focusing on the five dimensions, the mean score for global satisfaction was 4.7 out of 5, costs 4.6, medical services 4.7, privacy protection 4.7, and communication 4.7.

We investigated the total satisfaction scores and the five-dimension subscores further Table 3. There were significant differences among treatment response subgroups in total satisfaction scores and subscores. Patients with marked improvement (*N* = 956) had the highest mean total satisfaction score (23.5) and those who were rated as “no change or worse” (*N* = 8) on discharge had the lowest total satisfaction (22.5, *P* = 0.003). Compared to patients who were rated as having “no change or worse”, those who were rated as having “marked improvement”, “improvement”, or “some improvement” on discharge had significantly higher total satisfaction scores (23.5, 23.4, 23.1 vs 22.5, *P* = 0.003), higher scores on global satisfaction (4.7, 4.6, 4.5 vs 4.4, *P* = 0.002), medical services (4.7, 4.7, 4.6 vs 4.5, *P* = 0.001), privacy protection (4.8, 4.7, 4.7 vs 4.6, *P* = 0.010) and doctor-patient communication (4.7, 4.6, 4.6 vs 4.4, P = 0.003).

**Table 3:** Total satisfaction score and dimension score in subgroups and association analysis (mean ± standard deviation)

Characteristics	Total satisfaction score	*P*-value	Global satisfaction score	*P*-value	Costs satisfaction score	*P*-value	Medical services satisfaction score	*P*-value	Privacy protection satisfaction score	*P*-value	Doctor-patient communication satisfaction score	*P*-value
Sex		0.378		0.271		0.986		0.265		0.257		0.547
Male	23.4 ± 2.4		4.7 ± 0.5		4.6 ± 0.7		4.7 ± 0.5		4.7 ± 0.5		4.7 ± 0.6	
Female	23.3 ± 2.5		4.7 ± 0.5		4.6 ± 0.7		4.7 ± 0.6		4.7 ± 0.6		4.7 ± 0.6	
Marital status		0.471		0.960		0.611		0.443		0.437		0.304
Married	23.4 ± 2.3		4.7 ± 0.5		4.6 ± 0.7		4.7 ± 0.5		4.7 ± 0.5		4.7 ± 0.5	
Single or others	23.3 ± 2.5		4.7 ± 0.5		4.5 ± 0.7		4.7 ± 0.6		4.7 ± 0.6		4.7 ± 0.6	
Education level		0.163		0.093		0.545		0.442		0.046*		0.325
Elementary school	23.8 ± 2.8		4.6 ± 0.6		4.5 ± 0.7		4.7 ± 0.6		4.7 ± 0.6		4.6 ± 0.6	
Middle school	23.3 ± 2.2		4.7 ± 0.5		4.6 ± 0.6		4.7 ± 0.5		4.7 ± 0.5		4.7 ± 0.5	
High school	23.3 ± 2.5		4.7 ± 0.5		4.5 ± 0.7		4.7 ± 0.5		4.7 ± 0.5		4.7 ± 0.6	
College or above	23.5 ± 2.4		4.7 ± 0.5		4.6 ± 0.7		4.7 ± 0.6		4.8 ± 0.6		4.7 ± 0.6	
Insurance coverage		0.981		0.637		0.166		0.792		0.981		0.329
Self-pay	23.3 ± 2.3		4.7 ± 0.5		4.5 ± 0.8		4.7 ± 0.5		4.7 ± 0.5		4.7 ± 0.5	
Others	23.3 ± 2.5		4.7 ± 0.5		4.6 ± 0.7		4.7 ± 0.5		4.7 ± 0.6		4.7 ± 0.6	
Hospitalization times		0.829		0.612		0.241		0.698		0.228		0.194
First psychiatric hospitalization	23.3 ± 2.4		4.7 ± 0.5		4.5 ± 0.7		4.7 ± 0.5		4.7 ± 0.5		4.7 ± 0.5	
Recurrent psychiatric admission	23.3 ± 2.5		4.7 ± 0.5		4.6 ± 0.7		4.5 ± 0.5		4.7 ± 0.6		4.7 ± 0.6	
Involuntary hospitalization		0.917		0.505		0.952		0.693		0.871		0.546
Yes	23.3 ± 2.5		4.7 ± 0.5		4.6 ± 0.7		4.7 ± 0.5		4.7 ± 0.6		4.7 ± 0.6	
No	23.3 ± 2.4		4.7 ± 0.5		4.6 ± 0.7		4.7 ± 0.5		4.7 ± 0.5		4.7 ± 0.6	
Psychiatric diagnosis		0.724		0.866		0.628		0.826		0.633		0.016*
Schizophrenia or related disorders	23.3 ± 2.5		4.7 ± 0.5		4.6 ± 0.7		4.7 ± 0.5		4.7 ± 0.6		4.6 ± 0.6	
Mood disorders	23.4 ± 2.4		4.7 ± 0.5		4.5 ± 0.7		4.7 ± 0.5		4.7 ± 0.5		4.7 ± 0.5	
Others	23.4 ± 2.3		4.7 ± 0.5		4.6 ± 0.6		4.7 ± 0.5		4.7 ± 0.5		4.7 ± 0.6	
Treatment response		0.003**		0.002**		0.289		0.001**		0.010*		0.003**
Marked improvement	23.5 ± 2.4		4.7 ± 0.5		4.6 ± 0.7		4.7 ± 0.5		4.8 ± 0.5		4.7 ± 0.6	
Improvement	23.4 ± 2.4		4.6 ± 0.6		4.5 ± 0.6		4.7 ± 0.6		4.7 ± 0.6		4.6 ± 0.6	
Somewhat improvement	23.1 ± 2.6		4.5 ± 0.5		4.6 ± 0.7		4.6 ± 0.6		4.7 ± 0.5		4.6 ± 0.7	
No change or worse	22.5 ± 4.1		4.4 ± 1.2		4.5 ± 0.9		4.5 ± 0.9		4.6 ± 0.7		4.4 ± 0.7	
Days of hospital stay^b^	0.0	0.489	0.005	0.848	0.007	0.771	0.002	0.921	−0.017	0.491	−0.009	0.715
GAF(R)^b^	0.0	0.277	0.0	0.631	0.0	0.915	0.0	0.105	0.0	0.192	0.1	0.001**
Age(R)^b^	0.0	0.084	0.0	0.403	0.0	0.069	0.0	0.596	0.0	0.549	0.0	0.218
Number of times in physical restraints (R)^b^	0.0	0.197	0.0	0.843	0.0	0.644	0.0	0.720	0.0	0.057	0.0	0.791
Number of seclusion times(R)^b^	−0.1	< 0.001**	0.0	0.064	0.0	0.137	−0.1	0.002**	0.1	< 0.001**	−0.1	< 0.001**
Number of psychological treatment sessions(R)^b^	0.1	0.007**	0.1	0.005**	0.1	0.005**	0.1	0.003**	0.1	0.002**	0.0	0.107
Number of ECT treatments(R)^a,b^	−0.0	0.048*	0.0	0.275	0.0	0.108	0.0	0.104	0.0	0.286	−0.1	0.022*

^a^ Electric convulsive treatment

^b^ R, Spearman’s correlation coefficient

Bold values: *P < 0.05; **P < 0.01

Based on a bivariate analysis, patient’s education levels were also associated with satisfaction scores, as patients with a college education or above reported a significantly higher privacy protection subscore than lower education levels (4.8 vs 4.7, 4.7, 4.7, *P* = 0.046). Patients with schizophrenia or related disorders had the lowest communication subscore (4.6), compared to patients with mood disorders or another diagnosis (4.7, 4.7, *P* = 0.016). The GAF score was positively associated with doctor-patient communication satisfaction scores (coefficient = 0.1, *P* = 0.001).

Number of seclusions while admitted was significantly associated with total satisfaction scores (coefficient = − 0.1, *P* < 0.001), medical services satisfaction scores (coefficient = − 0.1, *P* = 0.002), privacy protection satisfaction scores (coefficient = 0.1, P < 0.001) and doctor-patient communication satisfaction scores (coefficient = − 0.1, P < 0.001).

The number of psychological therapy sessions was positively associated with total satisfaction scores (coefficient = 0.1, *P* = 0.007), global satisfaction scores (coefficient = 0.1, *P* = 0.005), cost scores (coefficient = 0.1, P = 0.005), medical service scores (coefficient = 0.1, *P* = 0.003) and privacy protection scores (coefficient = 0.1, *P* = 0.002).

The number of ECT treatments was inversely associated with total satisfaction scores (coefficient = − 0.0, *P* = 0.048) and with doctor-patient communication satisfaction scores (coefficient = − 0.1, *P* = 0.022).

### Correlates of patient satisfaction in multilevel analysis

A multilevel analysis was used to examine associations between individual-level and institution-level factors and satisfaction scores. In the null model, the intra-class correlation [32] was 22.6%, so multilevel analysis was necessary.

As shown in Table 4, global satisfaction scores were positively associated with college or above education level (coefficient = 0.2, *P* = 0.002), having “marked improvement” (coefficient = 0.6, *P* = 0.004), “improvement” (coefficient = 0.5, *P* = 0.016), and “some improvement” in response to treatment (coefficient = 0.6, *P* = 0.010). Cost satisfaction subscores were positively associated with college or above education level (coefficient = 0.1, *P* = 0.039); privacy protection satisfaction subscores were positively associated with college or above education level (coefficient = 0.2, *P* = 0.003) and number of psychological therapy sessions (coefficient = 0.0, *P* = 0.046); doctor-patient communication satisfaction scores were positively associated with having had “marked improvement” (coefficient = 0.5, *P* = 0.025), “improvement” (coefficient = 0.5, *P* = 0.050), or “some improvement” in response to treatment (coefficient = 0.5, P = 0.050), and GAF score (coefficient = 0.0, *P* = 0.003).

**Table 4:** Factors associated with total satisfaction score and dimensions score: multilevel analysis

	Total satisfaction β^a^	*P*-value	Global satisfaction β	*P*-value	Costs satisfaction β	*P*-value	Medical services satisfaction β	*P*-value	Privacy protection satisfaction β	*P*-value	Doctor -patient communication satisfaction β	*P*-value
Sex (ref. female)	0.2	0.863	0.0	0.194	0.0	0.674	0.1	0.062	0.0	0.257	0.0	0.410
Marital status (ref. single)	0.0	0.965	0.0	0.317	0.0	0.910	0.0	0.568	0.0	0.587	0.0	0.980
Education level (ref. elementary school)												
Middle school	0.3	0.773	0.1	0.139	0.1	0.278	0.0	0.339	0.1	0.131	0.0	0.419
High school	0.3	0.768	0.1	0.089	0.0	0.658	0.1	0.288	0.1	0.088	0.1	0.300
College or above	0.6	0.519	0.2	0.002**	0.1	0.039*	0.1	0.060	0.2	0.003**	0.1	0.079
Insurance coverage (ref. self-pay)	0.1	0.939	0.0	0.824	0.1	0.187	0.0	0.850	0.0	0.534	0.0	0.903
Number of hospitalizations (ref. recurrent admission)	−0.1	0.934	−0.0	0.259	0.0	0.424	0.0	0.685	0.0	0.779	0.0	0.864
Involuntary hospitalization (ref. No)	0.0	0.981	0.0	0.747	0.0	0.803	0.0	0.708	0.0	0.708	0.0	0.317
Psychiatric diagnosis (ref. Others)												
Schizophrenia or related disorders	0.0	0.993	0.0	0.745	0.0	0.972	0.0	0.874	0.0	0.874	0.0	0.596
Mood disorders	0.0	0.981	0.0	0.585	0.0	0.655	0.0	0.733	0.0	0.317	0.1	0.078
Treatment response (ref. No change or worse)												
Marked improvement	1.7	0.093	0.6	0.004**	0.1	0.634	0.4	0.082	0.0	0.882	0.5	0.025*
Improvement	1.3	0.193	0.5	0.016*	0.1	0.699	0.3	0.190	0.0	0.828	0.5	0.050*
Somewhat improvement	1.8	0.069	0.6	0.010*	0.3	0.322	0.4	0.079	0.1	0.743	0.5	0.050*
Days of hospital stay	0.0	0.999	0.0	0.317	0.0	0.317	0.0	0.317	0.0	0.317	0.0	0.317
GAF	0.0	0.995	0.0	0.317	0.0	0.317	0.0	0.317	0.0	0.317	0.0	0.003**
Age	0.0	0.992	0.0	0.131	0.0	0.131	0.0	0.339	0.0	0.317	0.0	0.131
Number of times in physical restraints	0.0	0.988	0.0	0.317	0.0	0.424	0.0	0.617	0.0	0.453	0.0	0.072
Number of times in seclusion	0.0	0.977	0.0	0.649	0.0	0.225	0.0	0.237	0.0	0.928	0.0	0.644
Number of psychological treatment sessions	0.0	0.996	0.0	0.317	0.0	0.317	0.0	0.317	0.0	0.046*	0.0	0.317
Number of ECT treatments	−0.1	0.955	−0.0	0.110	0.0	0.134	0.0	0.072	0.0	0.317	0.0	0.131
Psychiatrist-bed ratio	13.2	< 0.001**	2.7	< 0.001**	3.4	< 0.001**	2.2	< 0.001**	2.4	< 0.001**	2.5	< 0.001**
Nurse-bed ratio	6.2	< 0.001**	1.1	< 0.001**	1.4	< 0.001**	1.1	< 0.001**	1.4	< 0.001**	1.3	< 0.001**
Psychologists- bed ratio	3.5	0.001**	0.5	0.035*	1.3	< 0.001**	0.5	0.028*	0.6	0.010*	0.5	0.047*

^a^β, standardized beta coefficient (β represents a change in the standard deviation in the satisfaction score resulting from a one standard deviation change in the independent variable). Bold values: *P < 0.05; **P < 0.01

Total satisfaction scores and all five-dimension subscores were positively associated with institution-level factors, including psychiatrist-bed ratio, nurse-bed ratio and psychologist-bed ratio.

## Discussion

This study aimed to explore psychiatric inpatients’ satisfaction and the clinical and institutional correlates with satisfaction. To the best of our knowledge, this is the first national, cross-sectional survey in China with a sample this large. Our findings showed that the overall levels of satisfaction among psychiatric inpatients in China were high, with a mean score of 23.3 out of 25. This satisfaction level, which was measured by a different tool from other studies, is in line with surveys of psychiatric inpatients from other countries, such as India [33], Thailand [34], Finland [35], Israel [36], and Nigeria [37]. It is also consistent with a few local surveys of Chinese samples [24, 25].

Patient satisfaction is often affected by patient expectation: The high patient satisfaction in our study may have been due to low patient expectations. Since low expectations are more easily met, patients are likely to be satisfied with their experience [38]. In the context of widespread discrimination against psychiatric patients and the poor public image of psychiatric hospitals, the patient expectation for mental health hospitals was assumed to be low [39]. Measurements of patient’s expectations on the day of admission would help clarify this relationship.

Our study identified important factors associated with psychiatric inpatient satisfaction, some of which are at the individual level and others at the institutional level. Some are modifiable and can be used to improve psychiatric services which may in turn improve patient satisfaction.

At the patient level, treatment response was the most important factor associated with global patient satisfaction and with doctor-patient communication satisfaction. Although the differences of satisfaction score among patients with different treatment responses were statistically significant, the differences might not be clinically significant and could be due to the large sample sizes in each treatment response group. Regarding patient satisfaction, its relationship to treatment response makes sense as improvement in the patient’s illness is usually the patient’s goal for being in the hospital. Additionally, it aligns with findings from other studies [19, 40]. Regarding the doctor-patient communication, reductions in certain symptoms and improved interpersonal functioning would allow the patient to perceive the doctor as more helpful. Alternatively, the doctors may relate better and feel less frustrated by those who are showing a treatment response.

We found that patients with higher education levels had higher scores on global satisfaction, cost satisfaction, and privacy protection satisfaction. The literature on this topic is somewhat mixed. For example, one study found education level was not significantly associated with psychiatric patients’ satisfaction [16], another one found successful education increases patient satisfaction and results in improved adherence to treatment and thus to a better outcome [8], yet a third study showed patient satisfaction was significantly associated with less education [2]. Possible explanations for this inconsistency include the different measures of satisfaction used and the different patient populations recruited within these studies.

The current survey found that the GAF score and the number of psychological therapy sessions were significantly associated with patient satisfaction. Specifically, GAF was associated with the doctor-patient communication subscore, while therapy sessions were associated with privacy protection satisfaction. To our surprise, we did not find associations between the other clinical characteristics measured and patient satisfaction. This is quite different from other published reports in other countries [2, 16, 21, 41, 42]. It is possible that the expectations of patients in China are different from other countries, due to the differences in socio-culture and healthcare systems [43]. As suggested by previous studies, health needs may vary by ethnic groups [44]. For example, Vietnamese patients reported they had received better general practice care than other ethnic groups, and they also reported lower expectations [38]. Another possible explanation for the lack of associations between several clinical factors and patient satisfaction could be due to the statistical method we used. The multilevel analysis is often considered more stringent in variable selection than classical statistical analysis [31].

We found a few significant factors contributing to patient satisfaction at the hospital level. These factors, while important, are simply reflecting how well a hospital is professionally staffed. As expected, the quality of healthcare providers in a hospital would contribute to higher levels of patient satisfaction. We found that psychiatrist-bed ratio, nurse-bed ratio, and psychologist-bed ratio were all positively associated with patients’ total satisfaction and all five subscores. This is consistent with the findings reported by another study [17]. As mental health staff/patient ratios increased, psychiatric patients may have a higher likelihood of receiving higher quality psychiatric services and more clinical attention overall. Our findings suggest that, especially for those with inadequate staffing, the first step to improve patient satisfaction is to improve the number and quality of healthcare providers.

### Limitations

There are a few limitations about this study that need to be acknowledged. First, we only focused on patient-related variables and institutional variables. We did not have data on the healthcare providers (e.g. the psychiatrists, psychologists, nurses, etc), therefore we are unable to examine if there was any association between patient satisfaction and healthcare provider-related variables. This should be explored further in future studies. Second, this survey was conducted in the psychiatric hospitals under the jurisdiction of the Ministry of Health (Health Section) located in the capital cities. As a result, the data may not be generalizable to hospitals outside of the health sections, or in district/county level hospitals, hospitals located in rural areas, or in general hospitals. Third, our study used a self-reported psychiatric inpatient satisfaction instrument, in which response bias cannot be excluded. Patients involved may have reported greater satisfaction than they actually felt, as they may have believed that higher scores were more acceptable. Another limitation is the distribution of the questionnaires at patient discharge. Although some research has shown that satisfaction scores are lower if data are collected at a patient’s home [45], it was recommended that the satisfaction scale be administered before discharge in order to increase our response rate [27]. Furthermore, this research did not measure patients’ expectations, which were believed to be related to patient satisfaction. Additionally, as a new scale for the assessment of satisfaction was used in this research, it is difficult to compare our findings with those from earlier studies.

## Conclusion

In conclusion, based on a nationwide survey of psychiatric inpatients in China, we found that our respondents were very satisfied overall with their treatment. Additionally, we identified some potential determinants of patient satisfaction in China. At the individual level, a better education and a better treatment response were both associated with higher satisfaction scores, while at the institutional level, factors, such as the adequacy of staffing, were also related to higher satisfaction scores. To optimize patients’ satisfaction, the Chinese government should provide more resources to hospitals so that they may hire more mental health professionals and offer more psychological therapy. Future studies of psychiatric inpatients’ satisfaction should pay more attention to the patients’ expectations at the time of admission and should establish whether the associations reported in this analysis are observed in longitudinal studies.
